# An artificial intelligence ultrasound system’s ability to distinguish benign from malignant follicular-patterned lesions

**DOI:** 10.3389/fendo.2022.981403

**Published:** 2022-10-31

**Authors:** Dong Xu, Yuan Wang, Hao Wu, Wenliang Lu, Wanru Chang, Jincao Yao, Meiying Yan, Chanjuan Peng, Chen Yang, Liping Wang, Lei Xu

**Affiliations:** ^1^Department of Ultrasonography, The Cancer Hospital of the University of Chinese Academy of Sciences (Zhejiang Cancer Hospital), Institute of Basic Medicine and Cancer, Chinese Academy of Sciences, Hangzhou, China; ^2^Ultrasound Branch, Zhejiang Society for Mathematical Medicine, Hangzhou, China; ^3^Key Laboratory of Head & Neck Cancer Translational Research of Zhejiang Province, Zhejiang Provincial Research Center for Cancer Intelligent Diagnosis and Molecular Technology, Hangzhou, China; ^4^Shanghai Tenth People’s Hospital, Tongji University School of Medicine, Shanghai, China; ^5^School of Mathematical Sciences, Zhejiang University, Hangzhou, China; ^6^Department of Ultrasound, The Second Affiliated Hospital of Zhejiang Chinese Medical University, Hangzhou, China; ^7^Group of Computational Imaging and Digital Medicine, Zhejiang Qiushi Institute for Mathematical Medicine, Hangzhou, China; ^8^Group of Intelligent Medical Devices, South and North Lake Institute for Medical Artificial Intelligence, Haiyan, China

**Keywords:** thyroid adenomas, adenocarcinomas, follicular, ultrasonography, artificial intelligence

## Abstract

**Objectives:**

To evaluate the application value of a generally trained artificial intelligence (AI) automatic diagnosis system in the malignancy diagnosis of follicular-patterned thyroid lesions (FPTL), including follicular thyroid carcinoma (FTC), adenomatoid hyperplasia nodule (AHN) and follicular thyroid adenoma (FTA) and compare the diagnostic performance with radiologists of different experience levels.

**Methods:**

We retrospectively reviewed 607 patients with 699 thyroid nodules that included 168 malignant nodules by using postoperative pathology as the gold standard, and compared the diagnostic performances of three radiologists (one junior, two senior) and that of AI automatic diagnosis system in malignancy diagnosis of FPTL in terms of sensitivity, specificity and accuracy, respectively. Pairwise t-test was used to evaluate the statistically significant difference.

**Results:**

The accuracy of the AI system in malignancy diagnosis was 0.71, which was higher than the best radiologist in this study by a margin of 0.09 with a p-value of 2.08×10^-5^. Two radiologists had higher sensitivity (0.84 and 0.78) than that of the AI system (0.69) at the cost of having much lower specificity (0.35, 0.57 versus 0.71). One senior radiologist showed balanced sensitivity and specificity (0.62 and 0.54) but both were lower than that of the AI system.

**Conclusions:**

The generally trained AI automatic diagnosis system can potentially assist radiologists for distinguishing FTC from other FPTL cases that share poorly distinguishable ultrasonographical features.

## Highlights

- The AI automatic diagnosis system exhibited higher accuracy and specificity than radiologists in malignancy diagnosis of FPTL.
- The AI automatic diagnosis system had more balanced performance than radiologists in diagnosis of FPTL cases.

## Introduction

Thyroid carcinoma is the most common endocrine tumor in endocrine system. There is growing evidence in support of an increase in the occurrence of thyroid cancer. Lim et al. (1) reported that thyroid cancer incidence increased, on average, 3.6% per year during 1974–2013. Papillary thyroid cancer (PTC) incidence increased for all stages at diagnosis. Overall and distant PTC incidence-based mortality increased respectively 1.1% and 2.9% per year during 1994–2013. The main approaches to identify suspicious thyroid nodules are high-frequency color doppler ultrasonography and ultrasound-guided fine needle aspiration cytology (FNAC) (2). The Thyroid Imaging Reporting and Data Systems proposed by the American College of Radiology (ACR TI-RADS), which is a globally accepted malignancy risk stratification system for classifying thyroid nodules on the basis of their features at ultrasonography (US) imaging (3) shows high accuracy in distinguishing benign and malignant thyroid nodules, and can effectively reduce unnecessary biopsy of thyroid nodules on a large scale (4). However, the malignant ultrasonic features and risk categories of thyroid nodules in ACR TI-RADS are mainly based on papillary thyroid carcinoma (PTC), which accounts for the vast majority of malignant samples.

Currently, there are no clear instructions about how to distinguish benign and malignant thyroid follicular tumors in ACR TI-RADS. Follicular thyroid carcinoma (FTC), accounting for 5%-10% of all thyroid cancer, is the second common thyroid carcinoma (5). Compared with the most commonly occurring malignant PTC, FTC is less prone to lymph node metastasis, but more likely to relapse and metastasize to lungs and bones. When recurrence or distant metastasis occurs, it indicates a poor prognosis. In addition, compared with PTC, FTC is more likely to be locally invasive (6). Thyroid lobectomy alone may be sufficient initial treatment for low-risk follicular carcinomas; however, the treatment team may choose total thyroidectomy to enable RAI therapy for low to intermediate risk patients’ follicular carcinomas (2). Therefore, an accurate diagnosis of FTC before the initial operation has a tremendous influence on the surgical procedure and prognosis.

It has been found that, FTC shares similar characteristics in both ultrasound images (7–10) and FNAC (11–14) to other follicular-patterned thyroid lesions (FPTL) such as thyroid follicular adenoma (FTA) and adenomatoid hyperplasia nodule (AHN), hampering the malignancy diagnosis and their differential diagnosis. The gold standard for preoperative diagnosis of thyroid nodules Fine Needle Aspiration (FNA) can only diagnose follicular tumors and cannot distinguish between benign and malignant nodules. The final diagnosis instead relies on the detection of capsule involvement and vascular invasion in the postoperative pathological examinations (15). How to improve the preoperative differentiation of benign and malignant thyroid follicular tumors has important practical significance.

Genetic testing can, in principle, help diagnose thyroid nodules. For FPTL cases specifically, role of RAS mutations may be relevant (16–18). However, the most common thyroid related oncogene, namely the BRAFV600E mutation, is poor for malignancy differentiation of follicular patterned tumors (19, 20). In addition, compared with noninvasive ultrasonography, genetic testing requires more invasive fine needle aspiration biopsy and is also more costly. The advancement of AI technologies and especially the development of deep learning algorithms has brought radiologists new tools during the clinical studies for disease detection and diagnosis (21). Developing ultrasound-based AI technologies for thyroid nodule diagnosis has a potential to reduce invasive examinations. Convolutional neural networks have also been applied to the automatic detection and diagnosis of thyroid nodules (22–26). However, to our humble knowledge, currently there has not been any study trying to apply deep learning technologies to diagnose malignant nodules among FPTL that have indistinguishable ultrasonographic and cytologic features.

In this study, we applied the software development kit (SDK) of a generally trained thyroid nodule diagnosis system as it is without retraining for malignancy prediction of FPTL that included retrospectively collected FTC, FTA and AHN. This system, trained on an unselected population of nodules as opposed to nodules known to have follicular pattern, is initialized using self-training with noisy student method on ImageNet database, and takes a specially defined focal loss function to resolve the problem of unbalanced sample distribution that is frequently occurring in medical data. Focal Loss (27) increases the weight of rare classes in the loss function, making the minimization of loss function more sensitive to these samples, which is helpful to improve the accuracy of rare classes. In addition, it uses a Sharpness-Aware Minimization (SAM) algorithm to simultaneously minimize loss value and loss sharpness to improve the generalizability of the model (28). In particular, SAM algorithm seeks parameters that lie in neighborhoods with uniformly low loss. We compared its diagnostic performance with that of the radiologists of different experience levels using common evaluation metrics such as sensitivity, specificity and accuracy as well as two-tailed paired t-tests to verify whether if any observed differences were statistically significant.

## Materials and methods

### Data summary

A total of 607 patients with FTC, FTA and AHN (699 nodules) with complete but anonymized clinical information who underwent preoperative ultrasonography and complete examinations pulled from the provincial database from Zhejiang Society for Mathematical Medicine, with data contributed by 7 member hospitals, in which 263 nodules from The Cancer Hospital of the University of Chinese Academy of Sciences (Zhejiang Cancer Hospital), were included in this study. The histopathological diagnoses of all FTC, FTA and AHN were determined surgically. In summary, our study included 167 cases of FTC (23.89%), 241 cases of FTA (34.48%) and 291 cases of AHN (41.63%).

### Ultrasound examinations by radiologists and AI software

One junior radiologist A with 10 years of working experience and two senior radiologists, radiologist B and radiologist C, both with 20 years of working experience in ultrasound diagnosis performed the clinical ultrasound examinations on patients without knowing their pathological outcomes.

The ultrasound images were first grouped according to the associated nodules and then analyzed independently by all radiologists and the SDK (version 2.3.1.5) of the AI-SONIC™ Thyroid system with software version 5.3.0.2 (DE-Medicum Petavoxels Co., Ltd), which was developed on the EfficientNet architecture (29) using the proprietary deep learning framework DE-Light, and the system returned the predicted malignancy probability value of each nodule in the ultrasound images. The maximum malignancies predicted from the images associated to each nodule were chosen as the nodule-specific malignancy scores by all the raters, i.e., the radiologists and the AI. The malignancy probability value ranges from 0 to 1, and the cut-off value for the AI system was set by maximizing the mean of the sensitivity and specificity curves. In this study, the cut-off value was set to 0.4 by the AI system according to the analysis in **Supplementary Figure 1**. If the probability value is ≥ 0.4, the nodule is diagnosed as malignant, otherwise benign.

For further analysis of the nodule cases for which the AI system made correct diagnoses but failed by at least two of the three radiologists participating in the evaluation comparison study, the three radiologists who participated in the evaluation comparison study were asked to assign the ultrasound features according to ACR TI-RADS standards after discussions side-by-side and reviewed those images with a washout time longer than 6 months. We computed the sum of weighted scores by the frequency of nodule cases according to the TI-RADS scoring system for each individual ultrasonographic feature to obtain the average characteristic profile of these nodules.

### Statistical analysis

To assess the performance of the AI-SONIC™ system, we computed the Receiver operating Characteristic (ROC) curve and used the Area Under the Curve (AUC) as the evaluation metric. In order to compare its diagnosis with that of the radiologists, we calculated the sensitivity, specificity and accuracy. In addition, two-tailed t-test and McNemar test were used to compute p values for statistical comparisons. In all analyses, a p value less than 0.05 was considered a statistically significant difference. Statistical analysis was performed using Python 3.8 (Python Software Foundation, Delaware, United States).

## Results

### Comparison between the AI system and radiologists of different experience levels

**Figure 1:**
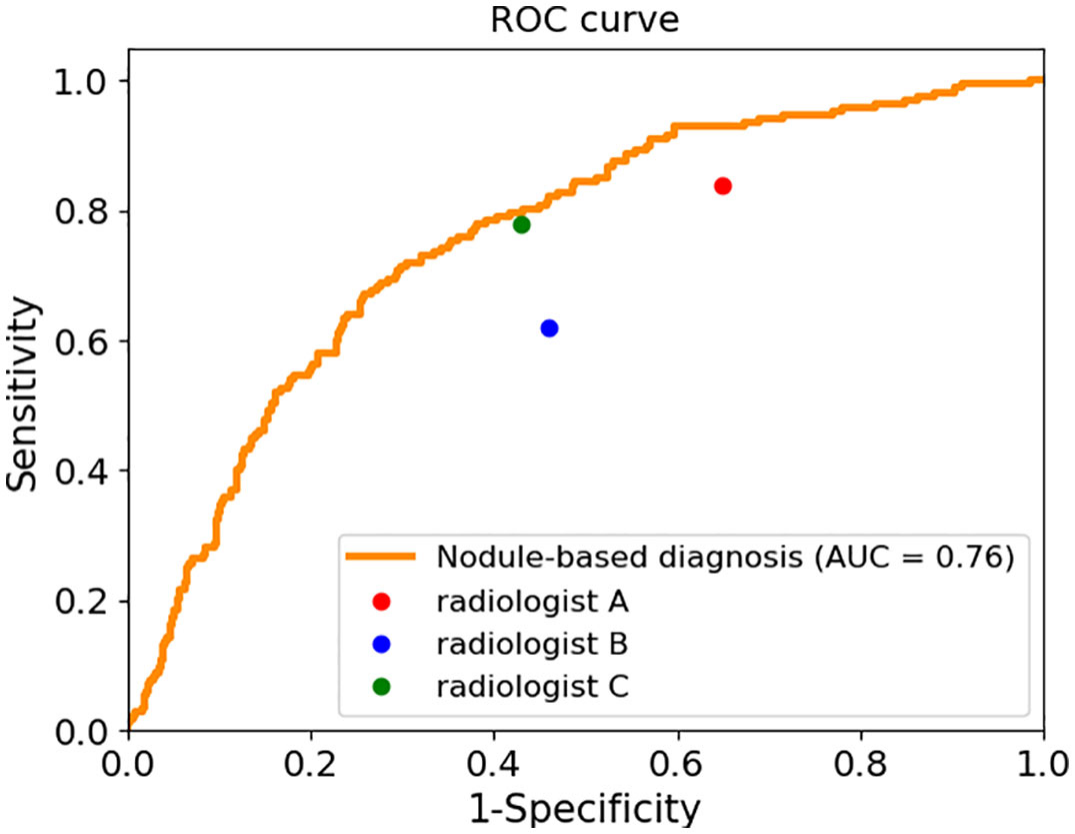
The sensitivity and specificity of three radiologists and the ROC curve and AUC value of the AI system.

**Figure 2:**
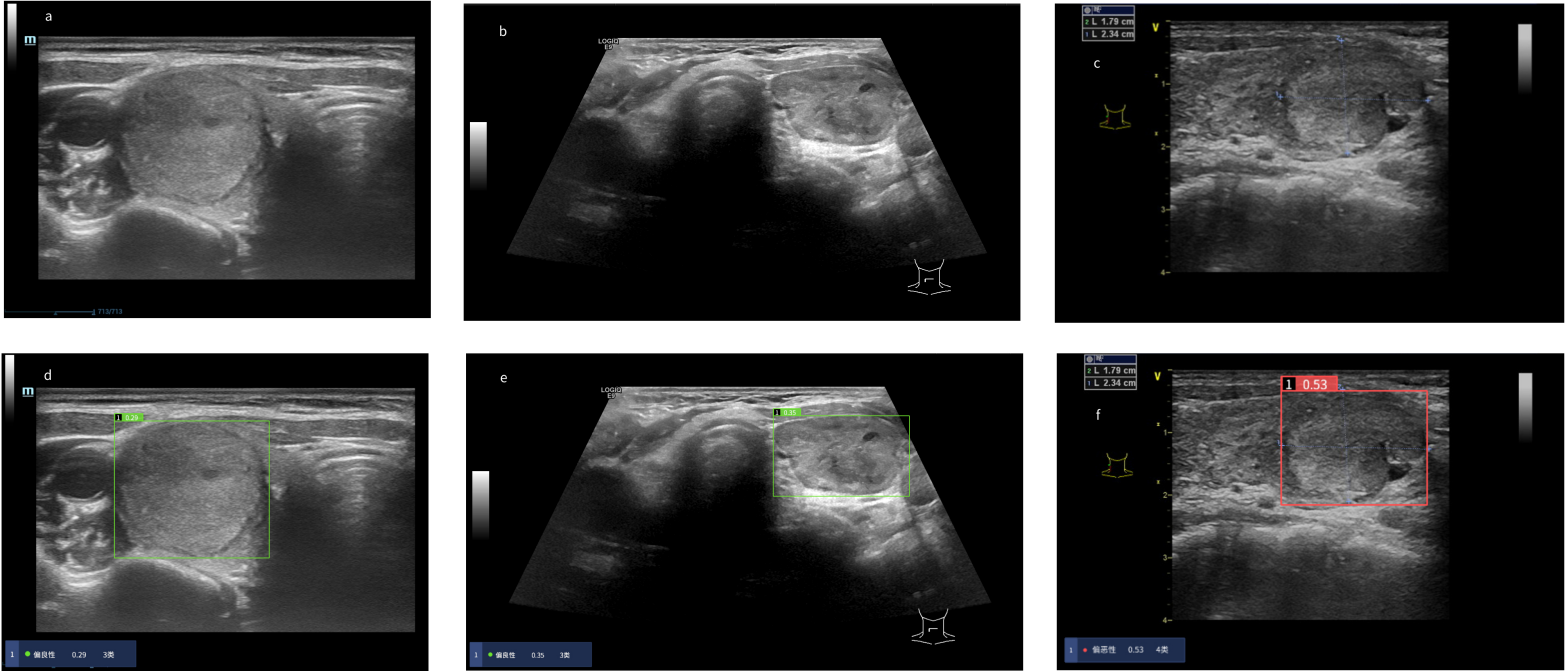
Risk coefficient assessment and diagnosis of thyroid nodules in the AI system. When the risk coefficient of thyroid nodules was < 0.4, the AI system diagnosed the thyroid nodules as “benign” as noted in the green display box, otherwise “malignant” as noted in the red display box. **(A–C)** Original ultrasound images of thyroid nodules. **(A)** Pathological diagnosis: thyroid follicular adenoma. **(B)** Pathological diagnosis: adenomatoid hyperplasia nodule. **(C)** Pathological diagnosis: follicular thyroid carcinoma. **(D–F)** Diagnosis of thyroid nodules in the AI system. **(D)** The AI system diagnosed the nodule as “benign”. Three radiologists diagnosed the nodule as “malignant”. **(E)** The AI system diagnosed the nodule as “benign”. Three radiologists diagnosed the nodule as “malignant”. **(F)** The AI system diagnosed the nodule as “malignant”. Three radiologists diagnosed the nodule as “benign”.

We calculated the sensitivity, specificity, positive predict value (PPV) and accuracy of the AI system and three radiologists in malignancy diagnosis of FPTL that consisted of FTC, FTA and AHN. The accuracy and PPV of the AI system was higher than all surveyed radiologists, as shown in **Table 1**. The sensitivity of the AI system however was lower than that of senior radiologist C (0.69 vs 0.78) and junior radiologist A (0.69 vs 0.84), but higher than that of senior radiologist B (0.69 vs 0.62). The specificity of the AI system in malignancy diagnosis was higher than all surveyed radiologists (0.71 vs 0.35, 0.54, 0.57 respectively). The overall performances were summarized in **Figure 1**, in which the ROC curve and the associated AUC value of the AI system were computed. Furthermore, we applied the McNemar test to compute p values between the AI system and the three radiologists. The results are as followes: p_AI-A_ = 1.71×10^-38^, p_AI-B_ = 2.10×10^-6^ and p_AI-C_ = 3.62×10^-10^. All p values between the AI system and three radiologists were less than 1×10^-5^. There were significant statistical differences between the AI system and three radiologists in diagnosing FTC, FTA and AHN. We presented a set of representative ultrasound images that showed the advantages of AI the system over radiologists in malignancy diagnosis of FPTL cases in **Figure 2**.

**Table 1:** The diagnostic performances of the AI system and three radiologists in thyroid malignancy nodules diagnosis.

	AI	Radiologist A (Junior)	Radiologist B (Senior)	Radiologist C (Senior)
Accuracy Sensitivity Specificity PPV	**0.71** 0.69 **0.71** **0.43**	0.47 **0.84** 0.35 0.29	0.56 0.62 0.54 0.30	0.62 0.78 0.57 0.36

Bold values are the highest values.

To further compare the diagnosis between the AI system and three radiologists, we subdivided the complete dataset to ten randomly divided subsets, summarized as in **Table 2**.

**Table 2:** The subdivided datasets for subsequent nodular diagnosis experiment.

Dataset	Total nodules	FTC	FTA	AHN
one	70	19	28	23
two	70	16	25	29
three	70	19	22	29
four	70	15	25	30
five	70	14	15	41
six	70	21	27	22
seven	70	15	23	32
eight	70	16	27	27
nine	70	12	23	35
ten	69	21	24	24

**Figure 3:**
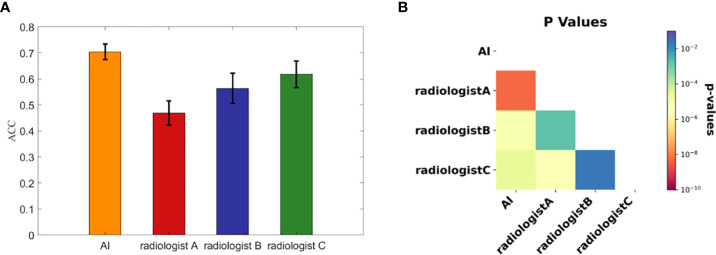
The diagnostic performances of three radiologists and the AI automatic diagnosis system. **(A)** The accuracies calculated from the ten subdivided datasets by the AI system and three radiologists. Each bar representing each concerned rater is presented with the average accuracy over the ten subdivided datasets and the standard deviation. **(B)** The associated p value matrix for statistical comparisons of diagnostic accuracies. All p values were <0.02 and self- comparisons were omitted as they were constant 1.

We calculated each rater’s accuracies in each dataset for malignancy diagnosis of FTC, FTA and AHN, and computed their average values and standard deviations over the ten datasets, with the corresponding results summarized in **Figure 3A**.

To statistically compare the diagnostic accuracies of the AI system and three radiologists in predicting thyroid nodule malignancies of FTC, FTA and AHN, we computed pairwise p values with the results shown in **Figure 3B**. Note that we skipped the statistical comparisons against oneselves, as in this case the p values were constant 1. All p values were < 0.02, where all p values between the AI system and three radiologists were less than 1×10^-4^. There were significant statistical differences between the AI system and three radiologists in diagnosing FTC, FTA and AHN.

### Comparison the performance of the AI system and radiologists in diagnosis FTC, FTA and AHN respectively

To further compare the AI system and three radiologists’ diagnosis, we calculated sensitivity and specificity for these three nodules, including FTC, FTA, and AHN, respectively. The results were shown in **Table 3**. When we only considered FTC cases, there were no true benign nodule cases in the numerator for the specificity calculation, resulting in a constant 0 for the specificity, which we omitted and showed only the sensitivity in the table. Similarly, for FTA and AHN cases, we presented only the specificity while ignored the sensitivity as there were no true malignant cases. For benign FPTL such as FTA and AHN, the specificity of the AI system was higher than that of the three radiologists. The sensitivity of the AI system was lower than that of senior radiologist C (0.69 vs 0.78) and junior radiologist A (0.69 vs 0.84), but higher than that of senior radiologist B (0.69 vs 0.62) in diagnosis FTC.

**Table 3:** The respective diagnostic performances of the AI system and three radiologists in diagnosing FTC, FTA and AHN cases.

	No. of cases	AI	Radiologist A(Junior)	Radiologist B(Senior)	Radiologist C(Senior)
**FTC**	167				
Sensitivity		0.69	**0.84**	0.62	0.78
**FTA**	241				
Specificity		**0.74**	0.34	0.51	0.5
**AHN**	291				
Specificity		**0.68**	0.37	0.57	0.63

When we only considered FTC cases, there were no true benign nodule cases, resulting in a constant 0 of the specificity, which we omitted in the table. Similarly, for FTA and AHN cases, we presented only the specificity while ignored the sensitivity for there were no true malignant cases.

Bold values are the highest values.

For further analysis, we selected the nodule cases for which the AI system made correct diagnoses but failed by at least two of the three radiologists participating in the evaluation comparison study and we got in total 144 benign and 12 malignant nodules. Our summarized results in **Table 4** show that for those 144 benign nodules, the sum of weighted scores (3.974) corresponds well to malignant suspicious category 4 nodules with an average characteristic profile of being predominantly solid, hypoechoic, with some but not pronounced echogenic foci and were considered by at least two radiologists to be malignant while they were correctly diagnosed to be benign by the AI system. For the 12 malignant nodules misdiagnosed by at least two radiologists to be benign, the sum of weight scores amounts to 3.916, with an average characteristic profile of being solid, mostly hypoechoic and predominantly without echogenic foci.

**Table 4:** Correlation of ultrasonographical features with nodular benignity and malignancy for which the AI system made correct diagnoses but failed by at least two of the three radiologists participating in the evaluation comparison study.

ACR TI-RADS Features	ACR Score	Benign (144)			Malignant (12)		
**Margin**		**Frequency**	**Probability**	**Weighted score**	**Frequency**	**Probability**	**Weighted score**
Smooth	0	120	0.833	0	6	0.500	0
Ill-defined	0	24	0.167	0	6	0.500	0
**Shape**							
Wider-than-tall	0	140	0.972	0	12	1.000	0
Taller-than-wide	3	4	0.028	0.084	0	0.000	0
**Echogenicity**							
Anechoic	0	1	0.007	0	0	0.000	0
Very hypoechoic	3	1	0.007	0.021	0	0.000	0
Hypoechoic	2	69	0.479	0.958	9	0.750	1.5
Isoechoic	1	72	0.500	0.5	3	0.250	0.25
Hyperechoic	1	1	0.007	0.007	0	0.000	0
**Composition**							0
Mixed cystic and solid	1	28	0.194	0.194	0	0.000	0
Cystic or almost completely cystic	0	1	0.007	0	0	0.000	0
Solid or almost completely solid	2	115	0.799	1.598	12	1.000	2
**Echogenic foci**							
Peripheral	2	8	0.056	0.112	1	0.083	0.166
Macro- calcifications	1	15	0.104	0.104	0	0.000	0
Punctate echogenic foci	3	19	0.132	0.396	0	0.000	0
None	0	102	0.708	0	11	0.917	0
**Sum**	–	144	1	3.974	12		3.916

## Discussion

FTC is a malignant follicular epithelial thyroid tumor with follicular differentiation but lacking the diagnosis characteristics of PTC. As previously noted, FTC has similar ultrasonic features to FTA and AHN, which have been identified by radiologists for differentiating malignant nodules from benign ones from a general perspective. And this is supported by this study that our three radiologists including two senior ones with experiences of more than 20 years in ultrasound diagnosis could at best reach an overall accuracy slightly more than 60% (62%) for malignancy predictions. It has been reported that the ultrasound diagnostic sensitivity for non-follicular thyroid tumors could reach 86.5%, but the diagnostic sensitivity for follicular tumors was only 18.2%, and the corresponding specificities for non-follicular and follicular tumors were 92.3% and 88.7% respectively (8). In our study, though the sensitivities in malignancy prediction by radiologists were all above 60%, the specificities could be as low as 35%. The poor diagnostic performance of the radiologists for FPTL cases can be most likely attributed to the fact that even senior radiologists lack diagnostic experiences due to the low overall incidence rate of thyroid follicular tumors. The AI-SONIC™ thyroid automatic diagnostic system which is trained for general benign and malignant nodule differentiation however provided a much better diagnostic accuracy of 0.71 for malignancy prediction of FPTL cases, and extremely balanced performance with the sensitivity and specificity being 0.69 and 0.71 respectively on a per-nodule analysis using the maximum malignancy scores computed from the images associated to each nodule. The p value based on two-tail pairwise t-test comparing the AI system and the best radiologist from ten randomly divided subsets in terms of malignancy accuracy in this study was 2.08×10^-5^, confirming the gap of 9% in accuracy was firmly statistically significant. In diagnosis of FTC, FTA and AHN respectively, the specificity of the AI system (0.74, 0.68) was higher than that of three radiologists, though the sensitivity of the AI system (0.69) was lower than the radiologist A (0.84) as well as radiologist C (0.78). However, the specificity of the radiologist A was extremely low (0.34, 0.37). This is probably because radiologist A could not distinguish the characteristics of benign and malignant FTPL, but had the tendency to overestimate the malignancy levels, resulting in high sensitivity and low specificity. Radiologist C could not differentiate between FTA and FTC. The AI system provided more balanced performance with the sensitivity and specificity. Our further analysis on the 144 benign cases where AI system made correct diagnoses but failed by at least two of the three radiologists suggests that the radiologists when using the TI-RADS scoring system might have a more conservative concern not to underplay the malignant potentials, consistent with radiologists’ higher sensitivity but lower specificity in thyroid nodule diagnosis. For the 12 malignant cases where AI system correctly diagnosed but failed by at least two of the three radiologists, since the number of these cases is small while being also at the boundary of being considered benign or malignant, it is difficult to assess whether this is significant. Nevertheless, the AI system can potentially assist radiologists distinguish FTC from other FPTL cases, given its higher overall accuracy and especially higher specificity. One possibility is to let the radiologists decide whether they would adopt the suggestions by the AI system or not, as long as higher diagnostic accuracy can be expected (30) from the AI system than the radiologists. Another possibility is to set up a rule so that a favorable outcome would be expected (26, 31). In our case, for instance, when the AI system predicts a nodule to be benign which is different from a radiologist’s decision given his or her assigned TI-RADS category, one lowers the category assignment by one. Overall, employing an algorithm or workflow that initially uses an AI diagnosis for classes of nodules for which it is superior to a radiologist, is more accurate than enabling a radiologist’s subjective decision to accept or reject it.

It shall be noted that in the context of benign thyroid nodular diseases, a high specificity of a diagnostic tool for malignancy detection is desirable. For inconclusive Bethesda categories that may be identified to be PTC follicular variant, noninvasive follicular thyroid neoplasm with papillary-like nuclear features (32), and FTC by histopathology, AI may help with benign and malignancy discrimination. Nevertheless, it was not anticipated that the AI system could manage well for distinguishing FTC from benign FPTL cases given that FTC has relatively rare incidence rate and that the design of the most widely applied ACR TI-RADS lexicon is based on the manifested malignant features of the most dominant PTC of all thyroid cancers. A reasonable explanation would be that the designer of the AI system has defined a focal loss function to resolve the problem of unbalanced sample distribution and likely has paid more attention to FPTL cases with higher learning weights. And the sharpness perception minimization algorithm that has been used could be beneficial for generalizability of their deep learning model.

It is interesting to point out that though FNAC is able to identify follicular tumors with high reliability, it has difficulty in predicting malignancy of FPTL cases (11–14). Therefore, it would be interesting to investigate whether applying the AI system for FPTL cases in combination with FNAC can help reduce the need of surgical excision for malignancy determination in the future. We have not included prospective data in this study because of the low prevalence of FPTL such that it can take a fairly long time to accumulate enough cases for statistically reliable evaluation. Apart from that, it would also be attractive to study how effective it is to train a deep learning model for differentiating follicular from papillary patterns of thyroid nodules based purely on retrospectively collected ultrasound images, which if good enough would help reduce unnecessary fine needle aspirations for future investigation.

The AI automatic diagnosis system can be potentially used as an auxiliary method for screening of FTC from other FPTL cases and may help reduce the need of surgical excision for further characterization given that FNAC has difficulty in determining the malignancy of these cases.

## Data availability statement

The data that support the findings of this study are available on reasonable request from corresponding author LW after formal approval by the concerned Chinese regulating authorities.

## Author contributions

Conceptualization, LW and LX; methodology, YW, JY and WL; software, YW, WL and WC; validation, LW and LX; formal analysis, YW and DX; investigation, HW, MY, CP and CY; resources, LW and LX; data curation, MY, CP and CY; writing—original draft preparation, WY and DX; writing—review and editing, LX; supervision, LW and LX; project administration, LW and LX; funding acquisition, DX and LW. All authors have read and agreed to the published version of the manuscript.

## Funding

This work was supported by the National Natural Science Foundation of China (No. 82071946) and the Zhejiang Provincial Natural Science Foundation of China (No. LY20H180001, LSD19H180001, LZY21F030001).

## Conflict of interest

The authors declare that the research was conducted in the absence of any commercial or financial relationships that could be construed as a potential conflict of interest.

## Publisher’s note

All claims expressed in this article are solely those of the authors and do not necessarily represent those of their affiliated organizations, or those of the publisher, the editors and the reviewers. Any product that may be evaluated in this article, or claim that may be made by its manufacturer, is not guaranteed or endorsed by the publisher.

## Supplementary material

Supplementary Figure 1 The changes of sensitivity and specificity with respect to the malignancy thresholds. **(A)** Evaluation metrics calculated from all 699 nodules, in which 168 were malignant. **(B)** Evaluation metrics calculated from randomly pulled 100 benign and 100 malignant nodules.

## Abbreviations

AI-CADx, Artificial Intelligent Computer- Aided Diagnosis System; AUC, Area Under the Curve; CNN, Convolutional Neural Network; FNAC, Fine Needle Aspiration Cytology; ROC, Receiver operating Characteristic; SDK, Software Development Kit; TI-RADS, Thyroid Imaging Reporting and Data System.
