# Supplementary material for: An artificial intelligence ultrasound system’s ability to distinguish benign from malignant follicular-patterned lesions
Source: Front Endocrinol (Lausanne). 2022 Oct 31;13:981403. doi: 10.3389/fendo.2022.981403 (PMC9660226; doi:10.3389/fendo.2022.981403)
Supplement: Supplementary Figure 1 — The changes of sensitivity and specificity with respect to the malignancy thresholds. (A) Evaluation metrics calculated from all 699 nodules, in which 168 were malignant. (B) Evaluation metrics calculated from randomly pulled 100 benign and 100 malignant nodules. [file DataSheet_1.doc]

**Appendix**

In order to choose the optimal cut-off value, we calculated curves of sensitivity and specificity with respect to the malignancy score threshold, shown in Supplementary Figure 1a. We maximized the mean of the sensitivity and specificity and the optimal cut-off value was found and rounded to be 0.4 in our experiment.

To test whether the cut-off value was distribution-dependent as in our study the benign cases clearly outnumbered the malignant cases given by the pathological examinations, we randomly selected 100 benign nodules and 100 malignant nodules and plotted the curves of sensitivity and specificity with respect to thresholds changes in Supplementary Figure 1b. It is clear that the trends of sensitivity and specificity were consistent with those computed for the entire dataset. Our results confirmed our cut-off value was distribution-independent.


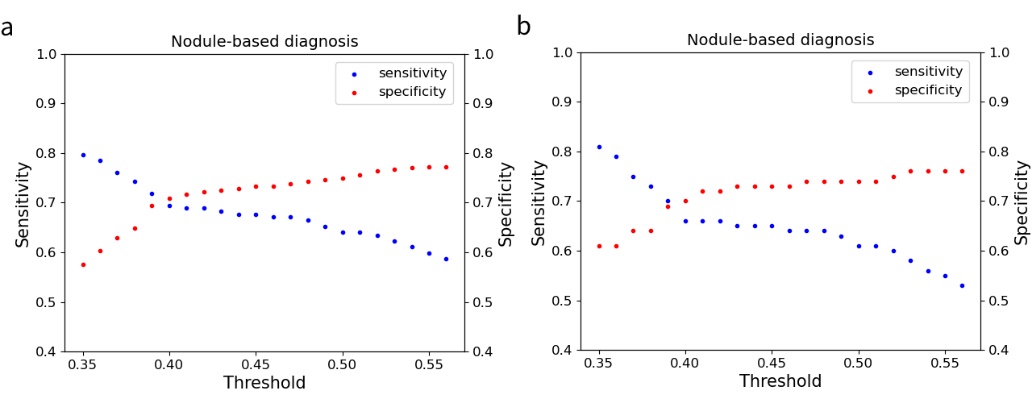


Supplementary Figure 1. The changes of sensitivity and specificity with respect to the malignancy thresholds. (a) evaluation metrics calculated from all 699 nodules, in which 168 were malignant. (b) evaluation metrics calculated from randomly pulled 100 benign and 100 malignant nodules.
